# Supplementary material for: An open label trial of anakinra to prevent respiratory failure in COVID-19
Source: eLife. 2021 Mar 8;10:e66125. doi: 10.7554/eLife.66125 (PMC8034977; doi:10.7554/eLife.66125)
Supplement: Supplementary file 1. [file elife-66125-supp1.docx]

**The SAVE Study**

Protocol and Statistical Analysis Plan

**Table of Contents**

| Content | Page |
| --- | --- |
| Final protocol (Version 3.0, 5^th^ October 2020) | 2 |
| Initial protocol (Version 1.0, 27^rd^ March 2020) | 30 |
| Statistical analysis plan | 58 |

**suPAR-GUIDED ANAKINRA TREATMENT FOR VALIDATION OF THE RISK AND EARLY MANAGEMENT OF SEVERE RESPIRATORY FAILURE BY COVID-19: THE SAVE OPEN-LABEL, NON-RANDOMIZED SINGLE-ARM TRIAL**

**STUDY PROTOCOL**

**Author: Evangelos J. Giamarellos-Bourboulis, MD, PhD**

**EudraCT number: 2020-001466-11**

**Protocol version: 3.0**

**Protocol date: 5 October 2020**

**Study Sponsor:**

**Hellenic Institute for the Study of Sepsis**

**88 Michalakopoulou Str., 115 28 Athens**

**e-mail:** [**insepsis@otenet.gr**](mailto:insepsis@otenet.gr)

**tel: +30 210 74 80 662**

**DISCLOSURE OF PRINCIPAL INVESTIGATOR**

**Protocol Study Title:** suPAR-GUIDED ANAKINRA TREATMENT FOR VALIDATION OF THE RISK AND EARLY MANAGEMENT OF SEVERE RESPIRATORY FAILURE BY COVID-19: THE SAVE OPEN-LABEL, NON-RANDOMIZED SINGLE-ARM TRIAL

The herein protocol became known to myself by the Study Sponsor. I understand that the protocol remains as yet unpublished; I certify that all disclosed information to myself for this protocol will remain strictly confidential.

The Principal Investigator,

Print Name

Signature Date

Table of Contents

[LIST OF ABBREVIATIONS 4](#_Toc51258644)

[SYNOPSIS 5](#_Toc51258645)

[BACKGROUND 7](#_Toc51258646)

[AIM OF THE STUDY 9](#_Toc51258647)

[STUDY DESIGN 9](#_Toc51258648)

[Study population 9](#_Toc51258649)

[Screening for eligibility 10](#_Toc51258650)

[Intervention 11](#_Toc51258651)

[Study drug 11](#_Toc51258652)

[Patients’ visits and interventions (Appendix II) 12](#_Toc51258653)

[LABORATORY PROCEDURES 16](#_Toc51258654)

[STUDY ENDPOINTS 16](#_Toc51258655)

[Primary study endpoint 16](#_Toc51258656)

[Secondary study endpoints 16](#_Toc51258657)

[NUMBER OF PATIENTS 17](#_Toc51258658)

INTERIM AND [STATISTICAL ANALYSIS 17](#_Toc51258659)

[ADVERSE EVENTS 18](#_Toc51258660)

[QUALITY CONTROL AND ASSURANCE 20](#_Toc51258661)

[ETHICAL CONSIDERATIONS 21](#_Toc51258662)

[PROTOCOL ADHERENCE AND AMENDMENTS 21](#_Toc51258663)

[REFERENCES 22](#_Toc51258664)

[APPENDIX I List of study sites 23](#_Toc51258665)

[APPENDIX II Study visits 25](#_Toc51258666)

[APPENDIX III The SOFA score 26](#_Toc51258667)

[APPENDIX IV Scoring of respiratory symptoms 27](#_Toc51258668)

[APPENDIX V Definition of severe respiratory failure 28](#_Toc51258669)

# LIST OF ABBREVIATIONS

AE: adverse event

CI: confidence interval

COVID-19: Coronavirus 2019 disease

CPAP: continuous positive airway pressure

EDTA: ethylene-diamene-tetracetic acid

FiO_2_: fraction of inspired oxygen

HR: hazard ratio

IL: interleukin

IFN: interferon

IV: intravenous

PBMCs: peripheral blood mononuclear cells

pO_2_: partial oxygen pressure

RCT: randomized clinical trial

SAE: serious adverse event

SRF: severe respiratory failure

SOFA: sequential organ failure assessment

suPAR: soluble urokinase plasminogen activator receptor

TNFα: tumor necrosis factor-alpha

# SYNOPSIS

| **Aim** | Recent data coming from the Hellenic Sepsis Study Group reveal that suPAR levels ≥6 ng/ml are early found among patients who will eventually develop SRF by COVID-19 with positive predictive value more than 80%. This signifies that an early pro-inflammatory reaction has been started in the lung. It is postulated that early anakinra treatment in these patients may halt this reaction and prevent development of SRF. |
| --- | --- |
| **Design** | Prospective, multicenter, open-label, single-arm trial |
| **Inclusion criteria** | - Age equal to or above 18 years - Male or female gender - In case of women, unwillingness to remain pregnant during the study period. - Written informed consent provided by the patient or by one first-degree relative/spouse in case of patients unable to consent - Confirmed infection by SARS-CoV-2 virus - Findings in chest-X-ray or in chest computed tomography compatible with lower respiratory tract infection - Plasma suPAR ≥6ng/ml |
| **Intervention** | Patients will receive once daily 100mg of anakinra subcutaneously for ten days. The drug should be administered on the same time ± 2 hours every day. All other administered drugs are allowed. In case the patient is discharged home before the completion of 10 days of treatment, it is at the discretion of the investigator to suggest treatment continuation at home. In case such a decision is taken, the patient will be provided the required number of pre-filled syringes for daily self-injection. |
| **Primary study endpoint** | The primary study endpoint is the rate of patients who will develop SRF (see appendix V for definition) until day 14. Patients dying before study visit of day 14 are considered achieving the primary endpoint. |
| **Secondary study endpoints** | - Comparison of the primary endpoint with comparators receiving standard-of-care treatment - Change of scoring for respiratory symptoms between days 1 and 7 - Change of scoring for respiratory symptoms between days 1 and 14 - Change of SOFA score between days 1 and 7 - Change of SOFA score between days 1 and 14 - Change of Peripheral Blood Mononuclear Cells’ (PBMCs) functionality between days 1 and 7 - Change of gene expression between days 1 and 7 (transcriptional, proteomic and metabolomic analysis) - Change of plasma inflammatory mediators between days 1 and 7 - 30-day mortality - 90-day mortality |
| **Number of patients** | The study power calculation is based on the hypothesis that 55% of patients with lower respiratory tract infection due to COVID-19 and suPAR≥6 ng/ml upon hospital admission, will develop SRF. To decrease this to 35% with power of 90% at a 5% level of significance, two hundred-sixty (260) patients need to be enrolled. Taking into consideration, the single-arm study design, 500 patients should be enrolled to make results more robust. |
| **Study duration** | 2 years |
| **Interim Analysis** | Interim analysis will be performed after completion of 30-day follow up of the first 130 patients. A comparison with a group of comparators receiving standard-of-care treatment will be performed. |

# BACKGROUND

The major hurdle of COVID-19 is the early recognition of the patients at high risk for the development of severe respiratory failure (SRF). If this can be achieved early, then appropriate immunomodulatory treatment may be administered to prevent development of SRF. This scenario is extremely visionary since it prevents the development of the major fatal consequence of Covid-19 but also alleviates the heavy medical and financial burden of Intensive Care Unit (ICU) admission.

Current evidence suggests that SARS-CoV-2 activates endothelial function which leads to over-production of D-dimers^1,2^. uPAR (urokinase plasminogen activator receptor) is anchored to the cell membranes of the lung endothelial cells. As result of the activation of kallikrein, uPAR is cleaved and enters the systemic circulation as the soluble counterpart suPAR^3^. Preliminary unpublished data from 57 Greek patients hospitalized after March 1^st^ 2020 in Greek hospitals due to pneumonia by confirmed SARS-CoV-2 infection showed that those with suPAR admission levels ≥ 6 ng/ml had greater risk for the development of SRF within 14 days than patients with suPAR less than 6ng/ml (Figure 1). The sensitivity of suPAR to detect these patients was 85.9% and the positive predictive value 85.9%. It needs to be outscored that all 21 Greek patients with suPAR≥ 6ng/ml were under standard-of-care treatment, which at that moment consisted of hydroxychloroquine and azithromycin. These data were confirmed in 15 patients hospitalized for pneumonia by SARS-CoV-2 in Rush Medical Center at Chicago^4^. A recent publication of 881 patients from the ISIC database verified these results^5^.

This prognostic ability of suPAR for unfavourable outcome is not presented for the first time; in the TRIAGE III trial that was conducted among 4,420 admissions in the emergency department in Denmark the interquartile range of suPAR was between 2.6 and 4.7 ng/ml in 30-day survivors and between 6.7 and 11.8 ng/ml in 30-day non-survivors^6^. Previous data from the Hellenic Sepsis Study Group on 1,914 patients clearly shows a high prognostic utility of admission suPAR for 28-day mortality^7^.


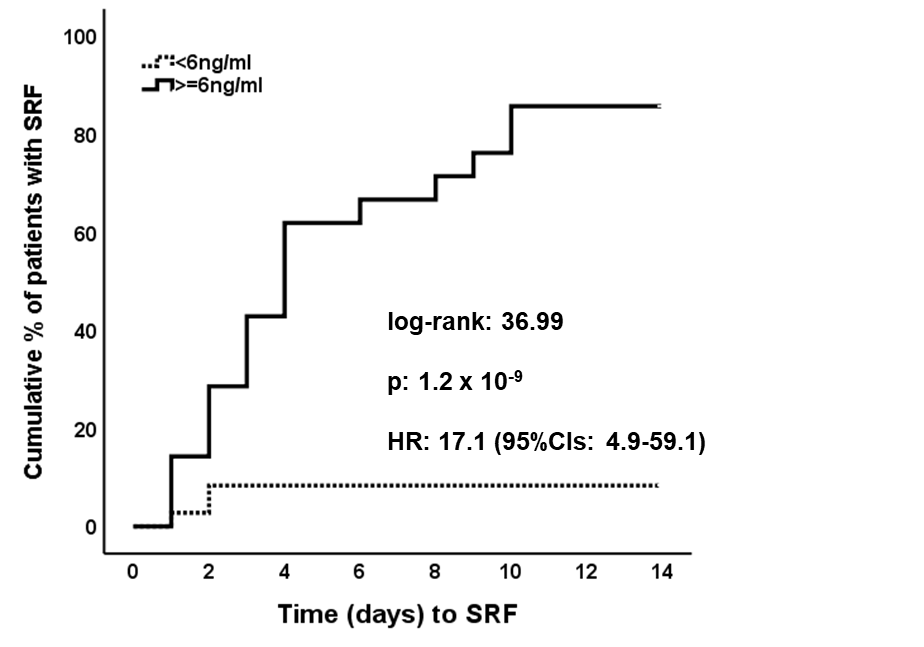


**Figure 1 Time to progression into SRF**

Patients are divided into those with suPAR ≥6 ng/ml on the day of hospital admission (n= 21) and into those with suPAR less than 6ng/ml on the day of hospital admission (n=36). The log-rank test and the p-value of comparison are provided

Abbreviations CI: confidence interval; HR: hazard ratio

It is obvious that suPAR can early identify the start of such a type of inflammatory process in the lung parenchyma that will soon be intensified. A recent publication has shown that this is due to the early release of IL-1α from lung epithelial cells that are infected by the virus. This IL-1α acts as a promoting factor that stimulates the production of IL-1β and of a further cytokine storm from alveolar macrophages^8^.

Anakinra is the only marketed product that inhibits both IL-1β and IL-1α and hence it is able to block an inflammatory response early on and to prevent the downstream inflammatory cascade. suPAR can be used as the biomarker tool to indicate patients with COVID-19 pneumonia in risk of SRF and in whom early start of anakinra may prevent development of SRF.

Anakinra is a safe drug that has been licensed for chronic subcutaneous administration in rheumatoid arthritis, refractory gout and chronic auto-inflammatory disorders^9^. The safety profile was further proven when it was administered in two randomized clinical trials where more than 1,500 critically ill patients with severe sepsis were intravenously treated^10,11^.

# AIM OF THE STUDY

In the SAVE study patients with lower respiratory tract infection by SARS-CoV-2 at high risk of SRF will be traced using suPAR. They will then start early treatment with anakinra in the aim to prevent the development of SRF.

# STUDY DESIGN

This will be a prospective open-label non-randomized study that will take place for 24 months in study sites in Greece (Appendix I). The study protocol will be approved by the National Ethics Committee of Greece and by the National Organization for Medicines of Greece. The study will be registered at Clinicaltrials.gov before enrolment of the first patient.

## Study population

Patients who meet ALL the following inclusion criteria and who do not meet any of the following exclusion criteria are allowed to be enrolled:

Inclusion criteria

1. Age equal to or above 18 years
2. Male or female gender
3. In case of women, unwillingness to remain pregnant during the study period.
4. Written informed consent provided by the patient or by one first-degree relative/spouse in case of patients unable to consent
5. Confirmed infection by SARS-CoV-2 virus
6. Findings in chest-X-ray or in chest computed tomography compatible with lower respiratory tract infection
7. Plasma suPAR ≥6ng/ml

Exclusion criteria

- Age below 18 years
- Denial for written informed consent
- Any stage IV malignancy
- Any do not resuscitate decision
- Presence of respiratory failure as defined in Appendix V
- Αny pO_2_/FiO_2_ ratio less than 150
- Any need for CPAP or mechanical ventilation
- Any primary immunodeficiency
- Less than 1,500 neutrophils/mm^3^
- Known hypersensitivity to anakinra
- Oral or IV intake of corticosteroids at a daily dose equal or greater than 0.4 mg prednisone for a period greater than the last 15 days.
- Any anti-cytokine biological treatment the last one month
- Severe hepatic failure
- Severe renal failure
- Pregnancy or lactation. Women of child-bearing potential will be screened by a urine pregnancy test before inclusion in the study

## Screening for eligibility

No study related procedure will be performed prior to obtaining written informed consent form. Screening follows these next steps:

- Step 1: The patient is screened for the exclusion criteria. If he meets any of them, he cannot be enrolled. If he does not meet any of them, he remains eligible and screening proceeds to Step 2
- Step 2: The patient is screened for inclusion criteria 1 to 6. If he meets these criteria, he remains eligible and screening proceeds to Step 3.
- Step 3: 18 ml of whole blood is drawn after venipuncture of one forearm vein under aseptic conditions and collected into one EDTA-coated and one non-EDTA-coated tube. The first tube is centrifuged for plasma collection. A commercialized quick blood test with suPARnostic® Quick Triage (Virogates S/A, Blokken 45, 3460 Birkerød, Denmark) will take place, to determinate in a very short time (20 min) suPAR levels in human EDTA-plasma. The sample (100 μl of plasma) will be incubated and handled, according to the manufacturer’s instructions, attached to a provided reader and the results will be displayed through LF Software. The measurement is the result of a lateral flow immunoassay (LFIA) and constitutes a quantitative measurement (in ng/ml) of plasma suPAR levels, provided that suPAR values are detected within the range of 2-15 ng/ml, to be considered accurate. If suPAR is found ≥6 ng/ml, the patient can be enrolled in the study. Blood analysis from blood collected in the second tube will be done as described at the section Laboratory Procedures

***Intervention***

Patients will receive 100mg of anakinra subcutaneously once daily for ten days. The drug should be administered on the same time ± 2 hours every day. In case the patient is discharged home before the completion of 10 days of treatment, it is at the discretion of the investigator to suggest treatment continuation at home. In case such a decision is taken, the patient will be provided the required number of pre-filled syringes for daily self-injection. In this case, the patient should return the empty used syringes within 30 days.

## Study drug

The active study drug i.e. anakinra will be provided in the form of pre-filled ready-to-use syringes. All syringes need to be stored at 2-8°C at the study site at a refrigerator with recording of temperature. In case recording indicates deviation of temperature below 0°C or above 10°C for more than a day, stored syringes need to be replaced by the Sponsor. At the exterior of each syringe there will be a letter and a 4-digit number. The letter refers to the study site, the first two digits of the number refer to the serial number of enrolled patient at the respective study site and the last two digits refer to the day of treatment. For example, the code A0102 refers to study site A, patient number 01 at that study site and treatment day 2. In case of patients discharged earlier and continuing the drug by self-injection, pre-filled syringes will be provided in a cooling bag.

The adverse events of anakinra are classified as very common (≥ 1/10), common (≥ 1/100 to < 1/10) and uncommon (≥ 1/1,000 to < 1/100) and they are presented in the Table below^7^:

| **MedDRA Organ System** | **Frequency** | **Undesirable Effect** |
| --- | --- | --- |
| Infections and infestations | Common | Serious infections |
| Blood and lymphatic system disorders | Common | Neutropenia  Thrombocytopenia |
| Immune system disorders | Uncommon | Allergic reactions including anaphylactic reactions, angioedema, urticaria and pruritus |
| Nervous system disorders | Very common | Headache |
| Hepatobiliary disorders | Uncommon | Hepatic enzyme increased |
|  | Not known | Non-infectious hepatitis |
| Skin and subcutaneous tissue disorders | Very common | Injection site reaction |
|  | Uncommon | Rash |
| Laboratory | Very common | Blood cholesterol increased |

## Patients’ visits and interventions (Appendix II)

*Day 1*

This visit will take place on the morning of the day of the start of treatment with the study drug. The following procedures will be done on that day:

- Recording of co-morbidities, co-administered drugs, past-history, SOFA score (Appendix III), absolute blood cell count and differential (if available) and biochemistry (if available)
- Scoring of the respiratory symptoms (Appendix IV).
- Administration of the study drug
- Recording of adverse events (AE) and severe adverse events (SAE)

*Day 2*

This visit will take place on the morning of the second day from the start of treatment with the study drug. The following procedures will be done on that day:

- Recording of co-administered drugs, absolute blood cell count and differential (if available) and biochemistry (if available)
- Recording of the presence or not of SRF (Appendix V)
- Recording of adverse events (AE) and severe adverse events (SAE)
- Administration of the study drug
- The visit may take place by phone call or through the internet in case of hospital discharge

*Day 3*

This visit will take place on the morning of the third day from the start of treatment with the study drug. The following procedures will be done on that day:

- Recording of co-administered drugs, absolute blood cell count and differential (if available) and biochemistry (if available)
- Recording of the presence or not of SRF (Appendix V)
- Recording of adverse events (AE) and severe adverse events (SAE)
- Administration of the study drug
- The visit may take place by phone call or through the internet in case of hospital discharge

*Day 4*

This visit will take place on the morning of the fourth day from the start of treatment with the study drug. The following procedures will be done on that day:

- Recording of co-administered drugs, absolute blood cell count and differential (if available) and biochemistry (if available)
- Recording of the presence or not of SRF (Appendix V)
- Recording of adverse events (AE) and severe adverse events (SAE)
- Administration of the study drug
- The visit may take place by phone call or through the internet in case of hospital discharge

*Day 5*

This visit will take place on the morning of the fifth day from the start of treatment with the study drug. The following procedures will be done on that day:

- Recording of co-administered drugs, absolute blood cell count and differential (if available) and biochemistry (if available)
- Recording of the presence or not of SRF (Appendix V)
- Recording of adverse events (AE) and severe adverse events (SAE)
- Administration of the study drug
- The visit may take place by phone call or through the internet in case of hospital discharge

*Day 6*

This visit will take place on the morning of the sixth day from the start of treatment with the study drug. The following procedures will be done on that day:

- Recording of co-administered drugs, absolute blood cell count and differential (if available) and biochemistry (if available)
- Recording of the presence or not of SRF (Appendix V)
- Recording of adverse events (AE) and severe adverse events (SAE)
- Administration of the study drug
- The visit may take place by phone call or through the internet in case of hospital discharge

*Day 7*

This visit will take place on the morning of the seventh day from the start of treatment with the study drug. The following procedures will be done on that day:

- Recording of co-administered drugs, SOFA score (Appendix III), absolute blood cell count and differential (if available) and biochemistry (if available)
- Scoring of the respiratory symptoms (Appendix IV).
- Recording of the presence or not of SRF (Appendix V)
- Recording of adverse events (AE) and severe adverse events (SAE)
- Sampling of 15 ml of venous blood. Blood analysis will be done as described at the section Laboratory Procedures.
- Administration of the study drug
- The visit may take place by phone call or through the internet in case of hospital discharge. In that case, no blood sampling will take place.

*Day 8*

This visit will take place on the morning of the eighth day from the start of treatment with the study drug. The following procedures will be done on that day:

- Recording of co-administered drugs, absolute blood cell count and differential (if available) and biochemistry (if available)
- Recording of the presence or not of SRF (Appendix V)
- Recording of adverse events (AE) and severe adverse events (SAE)
- Administration of the study drug
- The visit may take place by phone call or through the internet in case of hospital discharge

*Day 9*

This visit will take place on the morning of the nineth day from the start of treatment with the study drug. The following procedures will be done on that day:

- Recording of co-administered drugs, absolute blood cell count and differential (if available) and biochemistry (if available)
- Recording of the presence or not of SRF (Appendix V)
- Recording of adverse events (AE) and severe adverse events (SAE)
- Administration of the study drug
- The visit may take place by phone call or through the internet in case of hospital discharge

*Day 10*

This visit will take place on the morning of the tenth day from the start of treatment with the study drug. The following procedures will be done on that day:

- Recording of co-administered drugs, absolute blood cell count and differential (if available) and biochemistry (if available)
- Recording of the presence or not of SRF (Appendix V)
- Recording of adverse events (AE) and severe adverse events (SAE)
- Administration of the study drug
- The visit may take place by phone call or through the internet in case of hospital discharge

*Day 14*

This visit will take place on the morning of the 14^th^ day from the start of treatment with the study drug. The following procedures will be done on that day:

- Recording of co-administered drugs, SOFA score (Appendix III), absolute blood cell count and differential (if available) and biochemistry (if available)
- Scoring of the respiratory symptoms (Appendix IV).
- Recording of the presence or not of SRF (Appendix V)
- Recording of adverse events (AE) and severe adverse events (SAE)
- The visit may take place by phone call or through the internet in case of hospital discharge

# LABORATORY PROCEDURES

Blood samples will be collected in EDTA-coated and not-EDTA-coated tubes and in PAXgene tubes. Samples will be transferred on the day of collection to the central Lab, which is the the Laboratory of Immunology of Infections at the 4^th^ Department of Internal Medicine at ATTIKON University General Hospital. Analysis will be performed in the central Lab and in another European university Lab outside Greece.

Mediators of inflammation and suPAR will be measured in plasma and serum. Among measured mediators will be sIL-2R, IL-8 triglycerides as well as coagulation and fibrinolysis mediators.

All stored PAXgene tubes will be subjected to transcriptomic analysis. Part of the collected plasma will be stored at -80^o^C for proteomic and metabolomic analysis. Peripheral Blooed Mononuclear Cells (PBMCs) will be isolated after gradient centrifugation of whole blood over Ficoll. After serial washing, counting and exclusion of dead cells, they will be stimulated with purified ligands of SARS-CoV-2 for the production of TNFα, IL-1β, IL-6, ΙL-10, IL-17, IL-22 and IFNγ.

# STUDY ENDPOINTS

## Primary study endpoint

The primary study endpoint is the rate of patients who will develop SRF (see Appendix V for definition) until day 14. Patients dying before study visit of day 14 are considered achieving the primary endpoint.

## Secondary study endpoints

- Comparison of the primary endpoint with comparators receiving standard-of-care treatment
- Change of scoring for respiratory symptoms between days 1 and 7
- Change of scoring for respiratory symptoms between days 1 and 14
- Change of SOFA score between days 1 and 7
- Change of SOFA score between days 1 and 14
- Change of Peripheral Blood Mononuclear Cells’ (PBMCs) functionality between days 1 and 7
- Change of gene expression between days 1 and 7 (transcriptional, proteomic and metabolomic analysis)
- Change of plasma inflammatory mediators between days 1 and 7
- 30-day mortality
- 90-day mortality

# NUMBER OF PATIENTS

The study power calculation is based on the hypothesis that 55% of patients with lower respiratory tract infection due to COVID-19 and suPAR≥6 ng/ml upon hospital admission, will develop SRF. To decrease this to 35% with power of 90% at a 5% level of significance, two hundred sixty (260) patients need to be enrolled. Taking into consideration, the single-arm study design, 500 patients should be enrolled to make results more robust.

# INTERIM AND STATISTICAL ANALYSIS

An interim analysis will be conducted after completion of the 30-day follow up of the first 130 patients, i.e. 50% of the number of patients needed to be enrolled as calculated by power analysis. As comparators will be used patients hospitalized with pneumonia by the SARS-CoV-2 virus in other departments of Greek hospitals in which full data collection is performed by the Hellenic Sepsis Study Group, after written informed consent. Data collection in these departments has been approved by the Ethics Committees of the respective hospitals. For the purpose of this analysis, patients receiving standard-of-care treatment (SOC) will be selected among the total patients whose data are fully collected in order to fulfill the same inclusion and exclusion criteria as patients enrolled in the SAVE trial. Propensity matching will be performed among comparators and 130 patients enrolled in the SAVE trial, so as the 130 comparators will be fully comparable to all below criteria:

- Age
- Comorbidities, as expressed by the Charlson’s Comorbidity Index
- Admission severity scores namely pneumonia severity index (PSI), acute physiology and chronic health evaluation (APACHE) II score, SOFA score and WHO severity classification of COVID-19
- Co-administered treatment, such as azithromycin and dexamethasone

The primary endpoint will expressed as percentage and 95% confidence intervals. Comparisons with historical cases will be done by the Fisher exact test. Any p-value below 0.05 will be considered significant.

# ADVERSE EVENTS

Adverse events (AEs) and Serious Adverse Events (SAEs) will be collected from baseline until the last patient’s evaluation. Investigators should monitor subjects for adverse events and are responsible for recording ALL adverse events and serious adverse events occurring to a patient during the trial.

An adverse event is any undesirable medical occurrence in a subject administered a pharmaceutical product and which does not necessarily have a causal relationship with this treatment. The time relationship is defined from the moment the AE occurs during therapeutic treatment until 30 days or 5 half-lives after treatment discontinuation. The adverse event may be a sign, a symptom, or an abnormal laboratory finding.

**Serious adverse events** (SAEs) must be reported to within 24 hours. If an adverse event meets any of the following criteria, it is considered SAE:

- **Life-threatening situation** The subject was at risk of death at the time of the adverse event/experience. It does not refer to the hypothetical risk of death if the AE were more severe or were to progress.
- **Inpatient hospitalization** or prolongation of existing hospitalization.
- **Persistent or significant disability/incapacity** Any AE having an outcome that is associated with a substantial disruption of the ability to carry out normal life functions, including the ability to work. This is not intended to include transient interruption of daily activities.
- **Congenital anomaly/birth defects** Any structural abnormality in subject’s offspring that occurs after intrauterine exposure to treatment.
- **Important medical events/experiences** that may not result in death, be life-threatening, or require hospitalization may be considered a serious adverse event/experience when, based upon appropriate medical judgment, **they may jeopardize the subject and may require medical or surgical intervention to prevent one of the outcomes listed above,** i.e., death, a life-threatening adverse event/experience, inpatient hospitalization or prolongation of existing hospitalization, a persistent or significant disability/incapacity, or a congenital anomaly/birth defect. Examples of such medical events/experiences include allergic bronchospasm requiring intensive treatment in an emergency room or at home, blood dyscrasias or convulsions that do not result in inpatient hospitalization, or the development of drug dependency or drug abuse.
- **Spontaneous and elective abortions** experienced by study subject.

**A non-serious adverse event** is any untoward medical occurrence in a patient or subject who is administered a pharmaceutical product, and which does not necessarily have a causal relationship with this treatment. A non-serious adverse event is one that does not meet the definition of a serious adverse event given.

***Grading of severity***

The severity of the adverse events shall be graded as:

- **Mild** the adverse event is transient and well tolerated by the patient
- **Moderate** the adverse events causes discomfort and affects the usual activities of the patient.
- **Severe** the adverse events affects the usual activities of the patient to an important degree and may cause disability or be life-threatening.

***Relationship to the drug***

The investigator will use the following definitions to assess the relationship of the adverse event to study drug:

- **Probably Related**: The adverse event has a strong time relationship to the drug or relapses if re-induced, and another etiology is improbable or clearly less probable.
- **Possibly Related**: The adverse event has a strong time relationship to the drug and an alternative aetiology is as probable or less probable.
- **Probably not Related**: The adverse event has a slight or no time relationship to the drug and/or there is a more probable alternative aetiology.
- **Unrelated**: The adverse event is due to an underlying or concomitant disease or to another pharmaceutical product and is not related to the drug (no time relationship and a much more probable alternative aetiology).

If an investigator’s opinion of possibly related, probably not related or not related to study drug is given, an alternate etiology must be provided by the investigator. Please note that a severe adverse event/experience is not necessarily serious, as the term severe is a measure of intensity while a serious adverse event is determined based on the aforementioned regulatory criteria. Individual un-blinding thought to be necessary for the management of an adverse event will be documented in the subject Case Report Form.

# QUALITY CONTROL AND ASSURANCE

Quality control and assurance checks are performed by sponsor in order to allow periodic review of adequacy of the study activities and practices and allow for revising such practices as needed so the data and process are maintained, the study meets the protocol and procedural requirements, and is reproducible.

Before enrolling any subject in this study, sponsor personnel and the investigator have to review the protocol, the IB, the CRFs and instructions for their completion, the procedure for obtaining informed consent and the procedure for reporting AEs and SAEs.

A qualified representative of the sponsor will monitor the conduct of the study by visiting the site and by contacting the site by telephone and e-mail. During these site visits, all source documents are reviewed, and information recorded in the CRFs is verified against them.

Besides routine monitoring, quality assurance will be documented through independent auditing of the quality control activities and where applicable, by regulatory authorities through inspections.

**ETHICAL CONSIDERATIONS**

Prior to the initiation of this study, the study design will receive ethical, scientific, and where applicable, regulatory review. Investigators will conduct this study in accordance with the principles of the Declaration of Helsinki, GCP, and applicable regulatory requirements.

Regarding Informed Consent Form obtaining procedures, before any procedure specified in the protocol is performed, a subject must:

- Be informed of all pertinent aspects of the study and all elements of informed consent
- Be given time to ask questions and to consider the decision to participate
- Voluntarily agree to participate in the study
- Sign and date the updated and approved by IEC/REB ICF version.

**PROTOCOL ADHERENCE AND AMENDMENTS**

Investigators ascertain that they will apply due diligence to avoid protocol deviations. All significant protocol deviations will be recorded and reported in the clinical study report (CSR). Any change or addition to the protocol can only be made in a written protocol amendment that must be approved and signed by the sponsor, health authorities where required, and the IEC/REB.

# REFERENCES

1. Guan W, Ni Z, Hu Y, Liang W, Ou C, He J, et al. Clinical characteristics of coronavirus disease 2019 in China. *N Engl J Med* 2020; doi: 10.1056/ NEJMoa2002032
2. Huang C, Wang Y, Li X, Ren L, Zhao J, Hu Y, et al. [Clinical features of patients infected with 2019 novel coronavirus in Wuhan, China.](https://www.ncbi.nlm.nih.gov/pubmed/31986264) *Lancet* 2020; 395: 497-506
3. [Pixley RA](https://www.ncbi.nlm.nih.gov/pubmed/?term=Pixley%20RA%5BAuthor%5D&cauthor=true&cauthor_uid=21544310), [Espinola RG](https://www.ncbi.nlm.nih.gov/pubmed/?term=Espinola%20RG%5BAuthor%5D&cauthor=true&cauthor_uid=21544310), [Ghebrehiwet B](https://www.ncbi.nlm.nih.gov/pubmed/?term=Ghebrehiwet%20B%5BAuthor%5D&cauthor=true&cauthor_uid=21544310), [Joseph K](https://www.ncbi.nlm.nih.gov/pubmed/?term=Joseph%20K%5BAuthor%5D&cauthor=true&cauthor_uid=21544310), [Kao A](https://www.ncbi.nlm.nih.gov/pubmed/?term=Kao%20A%5BAuthor%5D&cauthor=true&cauthor_uid=21544310), [Bdeir K](https://www.ncbi.nlm.nih.gov/pubmed/?term=Bdeir%20K%5BAuthor%5D&cauthor=true&cauthor_uid=21544310), et al. Interaction of high-molecular-weight kininogen with endothelial cell binding proteins suPAR, gC1qR and cytokeratin 1 determined by surface plasmon resonance (BiaCore). [*Thromb Haemost*](https://www.ncbi.nlm.nih.gov/pubmed/21544310) 2011; 105: 1053-1059
4. Rovina N, Akinosoglou K, Eugen-Olsen E, Hayek S, Reiser J, Giamarellos-Bourboulis EJ. Soluble urokinase plasminogen activator receptor (suPAR)as an early predictor of severe respiratory failure in patients with COVID-19 pneumonia. *Crit Care* 2020; 24: 187
5. [Azam](https://pubmed.ncbi.nlm.nih.gov/?sort=date&term=Azam+TU&cauthor_id=32963090) TU, [Shadid](https://pubmed.ncbi.nlm.nih.gov/?sort=date&term=Shadid+HR&cauthor_id=32963090) HR, [Blakely](https://pubmed.ncbi.nlm.nih.gov/?sort=date&term=Blakely+P&cauthor_id=32963090) [P](https://pubmed.ncbi.nlm.nih.gov/32963090/#affiliation-1), [O'Hayer](https://pubmed.ncbi.nlm.nih.gov/?sort=date&term=O%27Hayer+P&cauthor_id=32963090) [P](https://pubmed.ncbi.nlm.nih.gov/32963090/#affiliation-2), [Berlin](https://pubmed.ncbi.nlm.nih.gov/?sort=date&term=Berlin+H&cauthor_id=32963090) H, [Pan](https://pubmed.ncbi.nlm.nih.gov/?sort=date&term=Pan+M&cauthor_id=32963090) [M](https://pubmed.ncbi.nlm.nih.gov/32963090/#affiliation-2), et al. Soluble urokinase receptor (SuPAR) in COVID-19-Related AKI, 2020; *J Am Soc Nephrol* doi: 10.1681/ASN.2020060829
6. [Schultz M](https://www.ncbi.nlm.nih.gov/pubmed/?term=Schultz%20M%5BAuthor%5D&cauthor=true&cauthor_uid=31236143), [Rasmussen LJH](https://www.ncbi.nlm.nih.gov/pubmed/?term=Rasmussen%20LJH%5BAuthor%5D&cauthor=true&cauthor_uid=31236143), [Høi-Hansen T](https://www.ncbi.nlm.nih.gov/pubmed/?term=H%C3%B8i-Hansen%20T%5BAuthor%5D&cauthor=true&cauthor_uid=31236143), [Kjøller E](https://www.ncbi.nlm.nih.gov/pubmed/?term=Kj%C3%B8ller%20E%5BAuthor%5D&cauthor=true&cauthor_uid=31236143), [Jensen BN](https://www.ncbi.nlm.nih.gov/pubmed/?term=Jensen%20BN%5BAuthor%5D&cauthor=true&cauthor_uid=31236143), [Lind MN](https://www.ncbi.nlm.nih.gov/pubmed/?term=Lind%20MN%5BAuthor%5D&cauthor=true&cauthor_uid=31236143), et al. Early discharge from the emergency department based on soluble urokinase plasminogen activator receptor (suPAR) levels: A TRIAGE III substudy. [*Dis Markers*](https://www.ncbi.nlm.nih.gov/pubmed/31236143) 2019; 2019: 3403549
7. Giamarellos-Bourboulis EJ, Norrby-Teglund A, Mylona V, Savva A, Tsangaris I, Dimopoulou I, et al. Risk assessment in sepsis: a new prognostication score by APACHE II score and serum soluble urokinase plasminogen activator receptor. *Crit Care* 2012; 16: R149
8. Conti P, Ronconi G, Caraffa A, Gallenga CE, Ross R, Frydas I, et al. [Induction of pro-inflammatory cytokines (IL-1 and IL-6) and lung inflammation by Coronavirus-19 (COVID-19 or SARS-CoV-2): anti-inflammatory strategies.](https://www.ncbi.nlm.nih.gov/pubmed/32171193) *J Biol Regul Homeost Agents* 2020; 34: doi: 10.23812/CONTI-E
9. Anakinra SmPC <https://www.ema.europa.eu/en/documents/product-information/kineret-epar-product-information_en.pdf>
10. Opal SM, Fisher CJ Jr, Dhainaut JF, Vincent JL, Brase R, Lowry SF, et al. [Confirmatory interleukin-1 receptor antagonist trial in severe sepsis: a phase III, randomized, double-blind, placebo-controlled, multicenter trial. The Interleukin-1 Receptor Antagonist Sepsis Investigator Group.](https://www.ncbi.nlm.nih.gov/pubmed/9233735) *Crit Care Med* 1997; 25: 1115-1122
11. Shakoory B, Carcillo JA, Chatham WW, Amdur RL, Zhao H, Dinarello CA, et al. [Interleukin-1 receptor blockade is associated with reduced mortality in sepsis patients with features of macrophage activation syndrome: reanalysis of a prior phase III trial.](https://www.ncbi.nlm.nih.gov/pubmed/26584195) *Crit Care Med* 2016; 44: 275-281

# APPENDIX I List of study sites

- 1^st^ Department of Internal Medicine, AHEPA University General Hospital of Thessaloniki (PI: Simeon Metallidis, Associate Professor of Internal Medicine and Infectious Diseases)
- 2^nd^ University Department of Internal Medicine, IPPOKRATEION General Hospital of Athens (PI: Helen Sambatakou, Assistant Professor of Internal Medicine and Infectious Diseases
- 3^rd^ University Department of Internal Medicine, General Hospital of Chest Diseases of Athens I SOTIRIA, (PI: Garyfallia Poulakou, Assistant Professor of Internal Medicine)
- Department of Internal Medicine, General Hospital of Chest Diseases of Athens I SOTIRIA (PI: Aikaterini Argyraki, Senior Registar)
- COVID-19 Department, General Hospital of Attica SISMANOGLEIO-AMALIA FLEMING, (PI: Malvina Lada, Director of NHS)
- Department of Internal Medicine, I PAMMAKARISTOS Hospital, (PI: Ioannis Baraboutis, Director of NHS)
- Department of Internal Medicine, University General Hospital of Patras PANAGIA I VOITHIA, (PI: Charalambos Gogos, Professor of Internal Medicine)
- Department of Internal Medicine, University General Hospital of Larissa, (PI: George Dalekos, Professor of Internal Medicine)
- 2^nd^ Department of Internal Medicine, University General Hospital of Alexandroupolis, (PI: Periklis Panagopoulos, Assistant Professor of Internal Medicine)
- Department of Clinical Therapeutics, ALEXANDRA General Hospital of Athens, (PI: Evangelos Kostis, Director of NHS)
- Department of Infectious Diseases, General Hospital of Kerkira, (PI: Ilias Papanikolaou, Senior Registar)
- 1^st^ University Department of Internal Medicine, General Hospital of Athens LAIKO, (PI: Michael Samarkos, Associate Professor of Internal Medicine and Infectious Diseases)
- 1^st^ Department of Internal Medicine, General Hospital of Athens G. GENNIMATAS, (PI: Georgios Adamis, Director of NHS)
- 1^st^ Department of Internal Medicine, General University Hospital of Ioannina, (PI: Charalampos Milionis, Professor of Internal Medicine)
- Department of Internal Medicine, General Hospital of Katerini (PI: Iraklis Tsanikidis, Director of NHS)
- 1^st^ Department of Internal Medicine, General Hospital of Athens KORGIALENIO-BENAKIO E.E.S. (PI: Tzavara Vasiliki, Director of NHS)
- 1^st^ University Department of Pulmonary Medicine, SOTIRIA General Hospital of Chest Diseases of Athens (PI: Professor Antonia Koutsoukou)
- 5^th^ Department of Pulmonary Medicine, SOTIRIA General Hospital of Chest Diseases of Athens (PI: Dimakou Aikaterini, Director of NHS)
- 1^st^ Department of Internal Medicine, General Hospital of Nea Ionia CONSTANTOPOULIO-PATISION (PI: Masgala-Seferli Aikaterini, Director of NHS)
- 1^st^ Department of Internal Medicine, General Hospital of Eleusis THRIASIO (PI: Symbardi Styliani, Director of NHS)
- 2^nd^ Department of Internal Medicine, General Hospital of Eleusis THRIASIO (PI: Alexiou Zoi, Director of NHS)
- Department of Internal Medicine, General Hospital of Athens ELPIS (PI: Fragkou Archontoula, Director of NHS)
- 3^rd^ Department of Internal Medicine, General Hospital of Athens KORGIALENEIO-BENAKEIO E.E.S. (PI: Chini Maria, Director of NHS)
- 1^st^ Department of Internal Medicine, General Hospital of Voula ASKLEPIEIO (PI: Bliziotis Ioannis, Director of NHS)
- 2^nd^ Department of Internal Medicine, General Hospital of Piraeus TZANEIO (PI: Chrysos Georgios, Director of NHS)
- Department of Internal Medicine, General Hospital of Larisa KOUTLIMBANEIO & ΤΡΙΑΝΤΑFΥLLΕΙΟ (PI: Karakoussis Konstantinos, Director of NHS)
- 2^nd^ Department of Internal Medicine, 251 Air Force General Hospital (PI: Symeonidis Nikolaos, Director)

**Monitor** Dr. Antigoni Kotsaki, MD, PhD

e-mail: [scra@sepsis.gr](mailto:scra@sepsis.gr) tel: +30 694 66 37 164; +30 210 58 32 562

**Pharmacovigilance supervisor** Ms Areti Voulomenou, MEng, MSc

e-mail: [voulomenou@suschem.gr](mailto:voulomenou@suschem.gr) tel +30 210 82 52 510

# APPENDIX II Study visits

| **Visits days** | **Screening** | **1** | **2** | **3** | **4** | **5** | **6** | **7** | **8** | **9** | **10** | **14** |
| --- | --- | --- | --- | --- | --- | --- | --- | --- | --- | --- | --- | --- |
| Informed consent | x |  |  |  |  |  |  |  |  |  |  |  |
| Exclusion criteria | x |  |  |  |  |  |  |  |  |  |  |  |
| Inclusion criteria | x |  |  |  |  |  |  |  |  |  |  |  |
| suPAR measurement | x |  |  |  |  |  |  | x |  |  |  |  |
| Scoring of symptoms |  | x |  |  |  |  |  | x |  |  |  | x |
| SOFA score |  | x |  |  |  |  |  | x |  |  |  | x |
| Blood sampling |  | x |  |  |  |  |  | x |  |  |  |  |
| Anakinra administration |  | x | x | x | x | x | x | x | x | x | x |  |
| Severe respiratory failure |  |  | x | x | x | x | x | x | x | x | x | x |
| AE/SAE |  | x | x | x | x | x | x | x | x | x | x | x |

# APPENDIX III The SOFA score

| **Variable** | **0 points** | **1 point** | **2 points** | **3 points** | **4 points** |
| --- | --- | --- | --- | --- | --- |
| PaO_2_/FiO_2_(mmHg) | ≥400 | <400 | <300 | <200 | <100 |
| Platelets (per mm^3^) | ≥150 | <150 | <100 | <50 | <20 |
| Hypotension | MAP≥ 70 mmHg | MAP<70 mmHg | Dobutamine whatever dose | Adrenaline≤0.1* or  Noradrenaline  ≤0.1* | Adrenaline>0.1* or  Noradrenaline >0.1* |
| Glasgow Coma Scale | 15 | 13-14 | 10-12 | 6-9 | <6 |
| Bilirubin (mg/dl) | <1.2 | 1.2-1.9 | 2.0-5.9 | 6.0-11.9 | ≥12 |
| Creatinine (mg/dl) or Urine output | <1.2 | 1.2-1.9 | 2.0-3.4 | 35-4.9 or <500ml/day | ≥5.0 or  <200ml/day |

*μg/kg/min

Each variable is scored between 0 and 4. The SOFA score is the sum of the score of each variable

# APPENDIX IV Scoring of respiratory symptoms

| **Symptom** | **Absent (score=0)** | **Mild (score=1)** | **Moderate (score=2)** | **Severe (score=3)** |
| --- | --- | --- | --- | --- |
| **Cough** | No cough or resolution (to pre-CAP levels) | Cough present but it does not interfere with subject’s usual daily activities | Cough present, frequent and it does interfere with some of the subject’s usual daily activities | Cough is present throughout the day and night; it limits most of the subject’s usual daily activities and sleep patterns |
| **Chest pain** | No chest pain or resolution of chest pain related to CAP | Chest pain present occasionally with deep breathing but it does not interfere with subject’s usual daily activities | Chest pain is present with normal breaths and it does interfere with the subject’s usual daily activities | Chest pain is present at rest and/or with shallow breathing; it limits most of the subject’s usual daily activities |
| **Shortness of breath (dyspnea)** | No shortness of breath or resolution (to pre-CAP Baseline) | Shortness of breath with strenuous activities only but it does not interfere with subject’s usual daily activities | Shortness of breath with usual activities and it does interfere with the subject’s usual daily activities | Shortness of breath with minimal exertion or at rest; it limits most of the subject’s usual daily activities |
| **Sputum** | No coughing up of phlegm/sputum or resolution (to pre-CAP Baseline) | Subject coughs up a small amount of phlegm/sputum | Subject coughs up a moderate amount of phlegm/sputum | Subject coughs up a large amount of phlegm/sputum |

# APPENDIX V Definition of severe respiratory failure

Presence of all of the following:

- pO_2_/FiO_2_ less than 150
- Need for mechanical or non-mechanical ventilation (CPAP)

**suPAR-GUIDED ANAKINRA TREATMENT FOR VALIDATION OF THE RISK AND EARLY MANAGEMENT OF SEVERE RESPIRATORY FAILURE BY COVID-19: THE SAVE OPEN-LABEL, NON-RANDOMIZED SINGLE-ARM TRIAL**

**STUDY PROTOCOL**

**Author: Evangelos J. Giamarellos-Bourboulis, MD, PhD**

**EudraCT number: 2020-001466-11**

**Protocol version: 1.0**

**Protocol date: 27 March 2020**

**Study Sponsor:**

**Hellenic Institute for the Study of Sepsis**

**88 Michalakopoulou Str., 115 28 Athens**

**e-mail:** [**insepsis@otenet.gr**](mailto:insepsis@otenet.gr)

**tel: +30 210 74 80 662**

**DISCLOSURE OF PRINCIPAL INVESTIGATOR**

**Protocol Study Title:** suPAR-GUIDED ANAKINRA TREATMENT FOR VALIDATION OF THE RISK AND EARLY MANAGEMENT OF SEVERE RESPIRATORY FAILURE BY COVID-19: THE SAVE OPEN-LABEL, NON-RANDOMIZED SINGLE-ARM TRIAL

The herein protocol became known to myself by the Study Sponsor. I understand that the protocol remains as yet unpublished; I certify that all disclosed information to myself for this protocol will remain strictly confidential.

The Principal Investigator,

Print Name

Signature Date

**Table of Contents**

[LIST OF ABBREVIATIONS 4](#_Toc45275552)

[SYNOPSIS 5](#_Toc45275553)

[BACKGROUND 7](#_Toc45275554)

[AIM OF THE STUDY 9](#_Toc45275555)

[STUDY DESIGN 9](#_Toc45275556)

[Study population 9](#_Toc45275557)

[Screening for eligibility 10](#_Toc45275558)

[Intervention 11](#_Toc45275559)

[Study drug 11](#_Toc45275560)

[Patients’ visits and interventions (Appendix II) 13](#_Toc45275561)

[LABORATORY PROCEDURES 17](#_Toc45275562)

[STUDY ENDPOINTS 18](#_Toc45275563)

[NUMBER OF PATIENTS 18](#_Toc45275564)

[STATISTICAL ANALYSIS 18](#_Toc45275565)

[ADVERSE EVENTS 18](#_Toc45275566)

[QUALITY CONTROL AND ASSURANCE 21](#_Toc45275567)

[ETHICAL CONSIDERATIONS 21](#_Toc45275568)

[PROTOCOL ADHERENCE AND AMENDMENTS 22](#_Toc45275569)

[REFERENCES 23](#_Toc45275570)

[APPENDIX I List of study sites 24](#_Toc45275571)

[APPENDIX II Study visits 26](#_Toc45275572)

[APPENDIX III The SOFA score 27](#_Toc45275573)

[APPENDIX IV Scoring of respiratory symptoms 28](#_Toc45275574)

[APPENDIX V Definition of severe respiratory failure 29](#_Toc45275575)

# LIST OF ABBREVIATIONS

AE: adverse event

CI: confidence interval

COVID-19: Coronavirus 2019 disease

CPAP: continuous positive airway pressure

EDTA: ethylene-diamene-tetracetic acid

FiO_2_: fraction of inspired oxygen

HR: hazard ratio

IL: interleukin

IFN: interferon

IV: intravenous

PBMCs: peripheral blood mononuclear cells

pO_2_: partial oxygen pressure

RCT: randomized clinical trial

SAE: serious adverse event

SRF: severe respiratory failure

SOFA: sequential organ failure assessment

suPAR: soluble urokinase plasminogen activator receptor

TNFα: tumor necrosis factor-alpha

# SYNOPSIS

| **Aim** | Recent data coming from the Hellenic Sepsis Study Group reveal that suPAR levels ≥6 ng/ml are early found among patients who will eventually develop SRF by COVID-19 with positive predictive value more than 80%. This signifies that an early pro-inflammatory reaction has been started in the lung. It is postulated that early anakinra treatment in these patients may halt this reaction and prevent development of SRF. |
| --- | --- |
| **Design** | Prospective, multicenter, open-label, single-arm trial |
| **Inclusion criteria** | - Age equal to or above 18 years - Male or female gender - In case of women, unwillingness to remain pregnant during the study period. - Written informed consent provided by the patient or by one first-degree relative/spouse in case of patients unable to consent - Confirmed infection by SARS-CoV-2 virus - Findings in chest-X-ray or in chest computed tomography compatible with lower respiratory tract infection - Plasma suPAR ≥6ng/ml |
| **Intervention** | Patients will receive once daily 100mg of anakinra subcutaneously and 80mg/400mg trimethoprim/sulfamethoxazole orally for ten days. The drug should be administered on the same time ± 2 hours every day. All other administered drugs are allowed. In case the patient is discharged home before the completion of 10 days of treatment, it is at the discretion of the investigator to suggest treatment continuation at home. In case such a decision is taken, the patient will be provided the required number of pre-filled syringes for daily self-injection. |
| **Primary study endpoint** | The primary study endpoint is the rate of patients who will not develop SRF (see appendix V for definition) until day 14. Patients dying before study visit of day 14 are considered non-achieving the primary endpoint. |
| **Secondary study endpoints** | - Comparison of the primary endpoint with historical comparators - Change of scoring for respiratory symptoms between days 1 and 7 - Change of scoring for respiratory symptoms between days 1 and 14 - Change of SOFA score between days 1 and 7 - Change of SOFA score between days 1 and 14 - Change of cytokine stimulation between days 1 and 7 - Change of plasma inflammatory mediators between days 1 and 7 |
| **Number of patients** | Although this is an exploratory trial, it is powered based on data from Greek patients showing that the 85.9% of those with lower respiratory tract infection by COVID-19 and suPAR≥6 ng/ml will develop SRF. To decrease this to 50% with power 80% at the 10% level of significance, sixty (60) patients need to be enrolled. Taking into consideration, the single-arm study design, 100 patients will be enrolled to make result more robust. |
| **Study duration** | 2 years |

# BACKGROUND

The major hurdle of COVID-19 is the early recognition of the patients at high risk for the development of severe respiratory failure (SRF). If this can be achieved early, then appropriate immunomodulatory treatment may be administered to prevent development of SRF. This scenario is extremely visionary since it prevents the development of the major fatal consequence of Covid-19 but also alleviates the heavy medical and financial burden of Intensive Care Unit (ICU) admission.

Current evidence suggests that SARS-CoV-2 activates endothelial function which leads to over-production of D-dimers^1,2^. uPAR (urokinase plasminogen activator receptor) is anchored to the cell membranes of the lung endothelial cells. As result of the activation of kallikrein, uPAR is cleaved and enters the systemic circulation as the soluble counterpart suPAR^3^. Preliminary unpublished data from 57 Greek patients hospitalized after March 1^st^ 2020 in Greek hospitals due to pneumonia by confirmed SARS-CoV-2 infection showed that those with suPAR admission levels ≥ 6 ng/ml had greater risk for the development of SRF within 14 days than patients with suPAR less than 6ng/ml (Figure 1). The sensitivity of suPAR to detect these patients was 85.9% and the positive predictive value 85.9%. It needs to be outscored that all 21 Greek patients with suPAR≥ 6ng/ml were under treatment with hydroxychloroquine and azithromycin. These data were confirmed in 15 patients hospitalized for pneumonia by SARS-CoV-2 in Rush Medical Center at Chicago (Rovina et al. submitted).

This prognostic ability of suPAR for unfavourable outcome is not presented for the first time; in the TRIAGE III trial that was conducted among 4,420 admissions in the emergency department in Denmark the interquartile range of suPAR was between 2.6 and 4.7 ng/ml in 30-day survivors and between 6.7 and 11.8 ng/ml in 30-day non-survivors^4^. Previous data from the Hellenic Sepsis Study Group on 1,914 patients clearly shows a high prognostic utility of admission suPAR for 28-day mortality^5^.


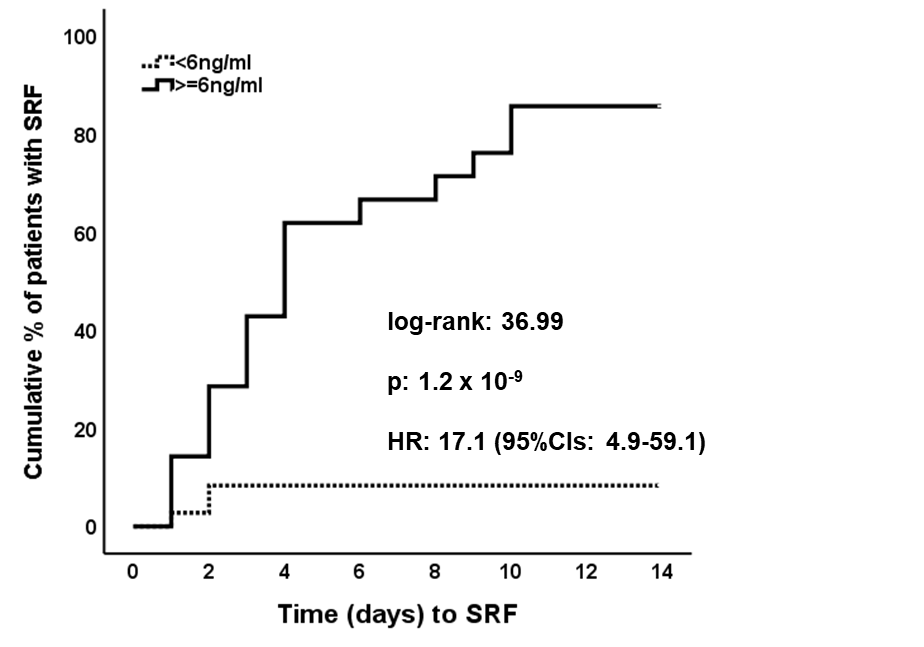


**Figure 1 Time to progression into SRF**

Patients are divided into those with suPAR ≥6 ng/ml on the day of hospital admission (n= 21) and into those with suPAR less than 6ng/ml on the day of hospital admission (n=36). The log-rank test and the p-value of comparison are provided

Abbreviations CI: confidence interval; HR: hazard ratio

It is obvious that suPAR can early identify the start of such a type of inflammatory process in the lung parenchyma that will soon be intensified. A recent publication has shown that this is due to the early release of IL-1α from lung epithelial cells that are infected by the virus. This IL-1α acts as a promoting factor that stimulates the production of IL-1β and of a further cytokine storm from alveolar macrophages^6^.

Anakinra is the only marketed product that inhibits both IL-1β and IL-1α and hence it is able to block an inflammatory response early on and to prevent the downstream inflammatory cascade. suPAR can be used as the biomarker tool to indicate patients with COVID-19 pneumonia in risk of SRF and in whom early start of anakinra may prevent development of SRF.

Anakinra is a safe drug that has been licensed for chronic subcutaneous administration in rheumatoid arthritis, refractory gout and chronic auto-inflammatory disorders^7^. The safety profile was further proven when it was administered in two randomized clinical trials where more than 1,500 critically ill patients with severe sepsis were intravenously treated^8,9^.

# AIM OF THE STUDY

In the SAVE study patients with lower respiratory tract infection by SARS-CoV-2 at high risk of SRF will be traced using suPAR. They will then start early treatment with anakinra in the aim to prevent the development of SRF.

# STUDY DESIGN

This will be a prospective open-label non-randomized study that will take place for 24 months in study sites in Greece (Appendix I). The study protocol will be approved by the National Ethics Committee of Greece and by the National Organization for Medicines of Greece. The study will be registered at Clinicaltrials.gov before enrolment of the first patient.

## Study population

Patients who meet ALL the following inclusion criteria and who do not meet any of the following exclusion criteria are allowed to be enrolled:

Inclusion criteria

1. Age equal to or above 18 years
2. Male or female gender
3. In case of women, unwillingness to remain pregnant during the study period.
4. Written informed consent provided by the patient or by one first-degree relative/spouse in case of patients unable to consent
5. Confirmed infection by SARS-CoV-2 virus
6. Findings in chest-X-ray or in chest computed tomography compatible with lower respiratory tract infection
7. Plasma suPAR ≥6ng/ml

Exclusion criteria

- Age below 18 years
- Denial for written informed consent
- Any stage IV malignancy
- Any do not resuscitate decision
- Absence of respiratory failure as defined in Appendix V
- Any primary immunodeficiency
- Less than 1,500 neutrophils/mm^3^
- Known hypersensitivity to anakinra
- Known hypersensitivity to trimethoprim/sulfamethoxazole
- Known G-6-PD enzyme deficiency
- Oral or IV intake of corticosteroids at a daily dose equal or greater than 0.4 mg prednisone for a period greater than the last 15 days.
- Any anti-cytokine biological treatment the last one month
- Pregnancy or lactation. Women of child-bearing potential will be screened by a urine pregnancy test before inclusion in the study

## Screening for eligibility

No study related procedure will be performed prior to obtaining written informed consent form. Screening follows these next steps:

- Step 1: The patient is screened for the exclusion criteria. If he meets any of them, he cannot be enrolled. If he does not meet any of them, he remains eligible and screening proceeds to Step 2
- Step 2: The patient is screened for inclusion criteria 1 to 6. If he meets these criteria, he remains eligible and screening proceeds to Step 3.
- Step 3: 3 ml of whole blood is drawn after venipuncture of one forearm vein under aseptic conditions and collected into one EDTA-coated tube. The tube is centrifuged for plasma collection. A commercialized quick blood test with suPARnostic® Quick Triage (Virogates S/A, Blokken 45, 3460 Birkerød, Denmark) will take place, to determinate in a very short time (20 min) suPAR levels in human EDTA-plasma. The sample (10 μl of plasma) will be incubated and handled, according to the manufacturer’s instructions, attached to a provided reader and the results will be displayed through LF Software. The measurement is the result of a lateral flow immunoassay (LFIA) and constitutes a quantitative measurement (in ng/ml) of plasma suPAR levels, provided that suPAR values are detected within the range of 2-15 ng/ml, to be considered accurate. If suPAR is found ≥6 ng/ml, the patient can be enrolled in the study.

## Intervention

Patients will receive 100mg of anakinra subcutaneously once daily for ten days. The drug should be administered on the same time ± 2 hours every day. In case the patient is discharged home before the completion of 10 days of treatment, it is at the discretion of the investigator to suggest treatment continuation at home. In case such a decision is taken, the patient will be provided the required number of pre-filled syringes for daily self-injection. In this case, the patient should return the empty used syringes within 30 days.

The patient will also receive in parallel to anakinra one oral table of 80mg/400mg trimethoprim/sulfamethoxazole as antimicrobial prophylaxis. The drug should be administered on the same time ± 2 hours every day. In case the patient is discharged home before the completion of 10 days of treatment, it is at the discretion of the investigator to suggest treatment continuation at home. In case such a decision is taken, the patient will be provided the required number of oral tablets of trimethoprim/sulfamethoxazole in a cartridge format. In this case, the patient should return the empty cartridges within 30 days.

All other administered drugs are allowed.

## Study drug

The active study drug i.e. anakinra will be provided in the form of pre-filled ready-to-use syringes. All syringes need to be stored at 2-8°C at the study site at a refrigerator with recording of temperature. In case recording indicates deviation of temperature below 0°C or above 10°C for more than a day, stored syringes need to be replaced by the Sponsor. At the exterior of each syringe there will be a letter and a 4-digit number. The letter refers to the study site, the first two digits of the number refer to the serial number of enrolled patient at the respective study site and the last two digits refer to the day of treatment. For example, the code A0102 refers to study site A, patient number 01 at that study site and treatment day 2. In case of patients discharged earlier and continuing the drug by self-injection, pre-filled syringes will be provided in a cooling bag.

The adverse events of anakinra are classified as very common (≥ 1/10), common (≥ 1/100 to < 1/10) and uncommon (≥ 1/1,000 to < 1/100) and they are presented in the Table below^7^:

| **MedDRA Organ System** | **Frequency** | **Undesirable Effect** |
| --- | --- | --- |
| Infections and infestations | Common | Serious infections |
| Blood and lymphatic system disorders | Common | Neutropenia  Thrombocytopenia |
| Immune system disorders | Uncommon | Allergic reactions including anaphylactic reactions, angioedema, urticaria and pruritus |
| Nervous system disorders | Very common | Headache |
| Hepatobiliary disorders | Uncommon | Hepatic enzyme increased |
|  | Not known | Non-infectious hepatitis |
| Skin and subcutaneous tissue disorders | Very common | Injection site reaction |
|  | Uncommon | Rash |
| Laboratory | Very common | Blood cholesterol increased |

The adverse events of trimethoprim/sulfamethoxazole are classified as very common (≥ 1/10), common (≥ 1/100 to < 1/10) and uncommon (≥ 1/1,000 to < 1/100) and they are presented in the Table below^10^:

| **MedDRA Organ System** | **Frequency** | **Undesirable Effect** |
| --- | --- | --- |
| Infections and infestations | Common | Overgrowth fungal |
|  | Very rare | Pseudomembranous colitis |
| Blood and lymphatic system disorders | Very rare | Leukopenia, neutropenia, thrombocytopenia, megaloblastic anaemia, aplastic anaemia, haemolytic anaemia, methaemoglobinaemia, eosinophilia, haemolysis in certain susceptible G-6-PD deficient patients. |
| Immune system disorders | Very rare | Serum sickness, anaphylactic reaction, allergic myocarditis, hypersensitivity vasculitis resembling Henoch-Schoenlein purpura, periarteritis nodosa, systemic lupus erythematosus. |
| Metabolism and nutrition disorders | Very common | Hyperkalaemia |
|  | Very rare | Hypoglycaemia, hyponatraemia, decreased appetite, metabolic acidosis |
| Psychiatric disorders | Very rare | Depression, hallucination. |
|  | Not known | Psychotic disorder |
| Nervous system disorders | Common | Headache. |
|  | Very rare | Aseptic meningitis, convulsions, neuropathy peripheral, ataxia, dizziness. |
| Ear and labrynth disorders | Very rare | Vertigo, tinnitus |
| Eye disorders | Very rare | Uveitis |
| Respiratory, thoracic and mediastinal disorders | Very rare | Cough, dyspnoea, lung infiltration |
| Gastrointestinal disorders | Common | Nausea, diarrhoea |
|  | Uncommon | Vomiting |
|  | Very rare | Glossitis, stomatitis, pancreatitis |
| Hepatobiliary disorders | Very rare | Increase of aminotransferases, bilirubin increase, cholestatic jaundice, hepatic necrosis |
| Skin and subcutaneous tissue disorders | Common | Rash |
|  | Very rare | Photosensitivity reaction, angiodema, dermatitis exfoliative, fixed drug eruption, erythema multiforme, Stevens-Johnson syndrome, toxic epidermal necrolysis |
|  | Not known | Drug reaction with eosinophilia and systemic symptoms (DRESS) |
| Musculoskeletal and connective tissue disorders | Very rare | Arthralgia, myalgia. |
| Renal and urinary disorders | Very rare | Renal impairment |

## Patients’ visits and interventions (Appendix II)

*Day 1*

This visit will take place on the morning of the day of the start of treatment with the study drug. The following procedures will be done on that day:

- Recording of co-morbidities, co-administered drugs, past-history, SOFA score (Appendix III), absolute blood cell count and differential (if available) and biochemistry (if available)
- Scoring of the respiratory symptoms (Appendix IV).
- Sampling of 15 ml of venous blood. This will be analyzed as described at the section Laboratory Procedures
- Administration of the study drugs
- Recording of adverse events (AE) and severe adverse events (SAE)

*Day 2*

This visit will take place on the morning of the second day from the start of treatment with the study drug. The following procedures will be done on that day:

- Recording of co-administered drugs, absolute blood cell count and differential (if available) and biochemistry (if available)
- Recording of the presence or not of SRF (Appendix V)
- Recording of adverse events (AE) and severe adverse events (SAE)
- Administration of the study drugs
- The visit may take place by phone call or through the internet in case of hospital discharge

*Day 3*

This visit will take place on the morning of the third day from the start of treatment with the study drug. The following procedures will be done on that day:

- Recording of co-administered drugs, absolute blood cell count and differential (if available) and biochemistry (if available)
- Recording of the presence or not of SRF (Appendix V)
- Recording of adverse events (AE) and severe adverse events (SAE)
- Administration of the study drugs
- The visit may take place by phone call or through the internet in case of hospital discharge

*Day 4*

This visit will take place on the morning of the fourth day from the start of treatment with the study drug. The following procedures will be done on that day:

- Recording of co-administered drugs, absolute blood cell count and differential (if available) and biochemistry (if available)
- Recording of the presence or not of SRF (Appendix V)
- Recording of adverse events (AE) and severe adverse events (SAE)
- Administration of the study drugs
- The visit may take place by phone call or through the internet in case of hospital discharge

*Day 5*

This visit will take place on the morning of the fifth day from the start of treatment with the study drug. The following procedures will be done on that day:

- Recording of co-administered drugs, absolute blood cell count and differential (if available) and biochemistry (if available)
- Recording of the presence or not of SRF (Appendix V)
- Recording of adverse events (AE) and severe adverse events (SAE)
- Administration of the study drugs
- The visit may take place by phone call or through the internet in case of hospital discharge

*Day 6*

This visit will take place on the morning of the sixth day from the start of treatment with the study drug. The following procedures will be done on that day:

- Recording of co-administered drugs, absolute blood cell count and differential (if available) and biochemistry (if available)
- Recording of the presence or not of SRF (Appendix V)
- Recording of adverse events (AE) and severe adverse events (SAE)
- Administration of the study drugs
- The visit may take place by phone call or through the internet in case of hospital discharge

*Day 7*

This visit will take place on the morning of the seventh day from the start of treatment with the study drug. The following procedures will be done on that day:

- Recording of co-administered drugs, SOFA score (Appendix III), absolute blood cell count and differential (if available) and biochemistry (if available)
- Scoring of the respiratory symptoms (Appendix IV).
- Recording of the presence or not of SRF (Appendix V)
- Recording of adverse events (AE) and severe adverse events (SAE)
- Sampling of 15 ml of venous blood. Blood analysis will be done as described at the section Laboratory Procedures.
- Administration of the study drugs
- The visit may take place by phone call or through the internet in case of hospital discharge. In that case, no blood sampling will take place.

*Day 8*

This visit will take place on the morning of the eighth day from the start of treatment with the study drug. The following procedures will be done on that day:

- Recording of co-administered drugs, absolute blood cell count and differential (if available) and biochemistry (if available)
- Recording of the presence or not of SRF (Appendix V)
- Recording of adverse events (AE) and severe adverse events (SAE)
- Administration of the study drugs
- The visit may take place by phone call or through the internet in case of hospital discharge

*Day 9*

This visit will take place on the morning of the nineth day from the start of treatment with the study drug. The following procedures will be done on that day:

- Recording of co-administered drugs, absolute blood cell count and differential (if available) and biochemistry (if available)
- Recording of the presence or not of SRF (Appendix V)
- Recording of adverse events (AE) and severe adverse events (SAE)
- Administration of the study drugs
- The visit may take place by phone call or through the internet in case of hospital discharge

*Day 10*

This visit will take place on the morning of the tenth day from the start of treatment with the study drug. The following procedures will be done on that day:

- Recording of co-administered drugs, absolute blood cell count and differential (if available) and biochemistry (if available)
- Recording of the presence or not of SRF (Appendix V)
- Recording of adverse events (AE) and severe adverse events (SAE)
- Administration of the study drugs
- The visit may take place by phone call or through the internet in case of hospital discharge

*Day 14*

This visit will take place on the morning of the 14^th^ day from the start of treatment with the study drug. The following procedures will be done on that day:

- Recording of co-administered drugs, SOFA score (Appendix III), absolute blood cell count and differential (if available) and biochemistry (if available)
- Scoring of the respiratory symptoms (Appendix IV).
- Recording of the presence or not of SRF (Appendix V)
- Recording of adverse events (AE) and severe adverse events (SAE)
- The visit may take place by phone call or through the internet in case of hospital discharge

# LABORATORY PROCEDURES

Blood samples will be used for the isolation of peripheral blood mononuclear cells (PBMCs) for cytokine stimulation and for the isolation of plasma.

PBMCs will be isolated after gradient centrifugation of whole blood over Ficoll. After serial washing, counting and exclusion of dead cells, they will be stimulated with purified ligands of SARS-CoV-2 for the production of TNFα, IL-1β, IL-6, ΙL-10, IL-17, IL-22 and IFNγ. Inflammatory mediators and suPAR will also be measured in patients’ plasma. Among measured mediators will be sIL-2R, IL-8 triglycerides and fibrinogen.

The study central lab will be the Laboratory of Immunology of Infections at the 4^th^ Department of Internal Medicine at ATTIKON University General Hospital.

# STUDY ENDPOINTS

*Primary study endpoint*

The primary study endpoint is the rate of patients who will not develop SRF (see Appendix V for definition) until day 14. Patients dying before study visit of day 14 are considered non-achieving the primary endpoint.

*Secondary study endpoints*

- Comparison of the primary endpoint with historical comparators
- Change of scoring for respiratory symptoms between days 1 and 7
- Change of scoring for respiratory symptoms between days 1 and 14
- Change of SOFA score between days 1 and 7
- Change of SOFA score between days 1 and 14
- Change of cytokine stimulation between days 1 and 7
- Change of plasma inflammatory mediators between days 1 and 7

# NUMBER OF PATIENTS

Although this is an exploratory trial, it is powered based on data from Greek patients showing that the 85.9% of those with lower respiratory tract infection by COVID-19 and suPAR≥6 ng/ml will develop SRF. To decrease this to 50% with power 80% at the 10% level of significance, sixty (60) patients need to be enrolled. Taking into consideration, the single-arm study design, 100 patients will be enrolled to make result more robust.

# STATISTICAL ANALYSIS

The primary endpoint will expressed as percentage and 95% confidence intervals. Comparisons with historical cases will be done by the Fisher exact test. Any p-value below 0.05 will be considered significant.

# ADVERSE EVENTS

Adverse events (AEs) and Serious Adverse Events (SAEs) will be collected from baseline until the last patient’s evaluation. Investigators should monitor subjects for adverse events and are responsible for recording ALL adverse events and serious adverse events occurring to a patient during the trial.

An adverse event is any undesirable medical occurrence in a subject administered a pharmaceutical product and which does not necessarily have a causal relationship with this treatment. The time relationship is defined from the moment the AE occurs during therapeutic treatment until 30 days or 5 half-lives after treatment discontinuation. The adverse event may be a sign, a symptom, or an abnormal laboratory finding.

**Serious adverse events** (SAEs) must be reported to within 24 hours. If an adverse event meets any of the following criteria, it is considered SAE:

- **Life-threatening situation** The subject was at risk of death at the time of the adverse event/experience. It does not refer to the hypothetical risk of death if the AE were more severe or were to progress.
- **Inpatient hospitalization** or prolongation of existing hospitalization.
- **Persistent or significant disability/incapacity** Any AE having an outcome that is associated with a substantial disruption of the ability to carry out normal life functions, including the ability to work. This is not intended to include transient interruption of daily activities.
- **Congenital anomaly/birth defects** Any structural abnormality in subject’s offspring that occurs after intrauterine exposure to treatment.
- **Important medical events/experiences** that may not result in death, be life-threatening, or require hospitalization may be considered a serious adverse event/experience when, based upon appropriate medical judgment, **they may jeopardize the subject and may require medical or surgical intervention to prevent one of the outcomes listed above,** i.e., death, a life-threatening adverse event/experience, inpatient hospitalization or prolongation of existing hospitalization, a persistent or significant disability/incapacity, or a congenital anomaly/birth defect. Examples of such medical events/experiences include allergic bronchospasm requiring intensive treatment in an emergency room or at home, blood dyscrasias or convulsions that do not result in inpatient hospitalization, or the development of drug dependency or drug abuse.
- **Spontaneous and elective abortions** experienced by study subject.

**A non-serious adverse event** is any untoward medical occurrence in a patient or subject who is administered a pharmaceutical product, and which does not necessarily have a causal relationship with this treatment. A non-serious adverse event is one that does not meet the definition of a serious adverse event given.

***Grading of severity***

The severity of the adverse events shall be graded as:

- **Mild** the adverse event is transient and well tolerated by the patient
- **Moderate** the adverse events causes discomfort and affects the usual activities of the patient.
- **Severe** the adverse events affects the usual activities of the patient to an important degree and may cause disability or be life-threatening.

***Relationship to the drug***

The investigator will use the following definitions to assess the relationship of the adverse event to study drug:

- **Probably Related**: The adverse event has a strong time relationship to the drug or relapses if re-induced, and another etiology is improbable or clearly less probable.
- **Possibly Related**: The adverse event has a strong time relationship to the drug and an alternative aetiology is as probable or less probable.
- **Probably not Related**: The adverse event has a slight or no time relationship to the drug and/or there is a more probable alternative aetiology.
- **Unrelated**: The adverse event is due to an underlying or concomitant disease or to another pharmaceutical product and is not related to the drug (no time relationship and a much more probable alternative aetiology).

If an investigator’s opinion of possibly related, probably not related or not related to study drug is given, an alternate etiology must be provided by the investigator. Please note that a severe adverse event/experience is not necessarily serious, as the term severe is a measure of intensity while a serious adverse event is determined based on the aforementioned regulatory criteria. Individual un-blinding thought to be necessary for the management of an adverse event will be documented in the subject Case Report Form.

# QUALITY CONTROL AND ASSURANCE

Quality control and assurance checks are performed by sponsor in order to allow periodic review of adequacy of the study activities and practices and allow for revising such practices as needed so the data and process are maintained, the study meets the protocol and procedural requirements, and is reproducible.

Before enrolling any subject in this study, sponsor personnel and the investigator have to review the protocol, the IB, the CRFs and instructions for their completion, the procedure for obtaining informed consent and the procedure for reporting AEs and SAEs.

A qualified representative of the sponsor will monitor the conduct of the study by visiting the site and by contacting the site by telephone and e-mail. During these site visits, all source documents are reviewed, and information recorded in the CRFs is verified against them.

Besides routine monitoring, quality assurance will be documented through independent auditing of the quality control activities and where applicable, by regulatory authorities through inspections.

**ETHICAL CONSIDERATIONS**

Prior to the initiation of this study, the study design will receive ethical, scientific, and where applicable, regulatory review. Investigators will conduct this study in accordance with the principles of the Declaration of Helsinki, GCP, and applicable regulatory requirements.

Regarding Informed Consent Form obtaining procedures, before any procedure specified in the protocol is performed, a subject must:

- Be informed of all pertinent aspects of the study and all elements of informed consent
- Be given time to ask questions and to consider the decision to participate
- Voluntarily agree to participate in the study
- Sign and date the updated and approved by IEC/REB ICF version.

**PROTOCOL ADHERENCE AND AMENDMENTS**

Investigators ascertain that they will apply due diligence to avoid protocol deviations. All significant protocol deviations will be recorded and reported in the clinical study report (CSR). Any change or addition to the protocol can only be made in a written protocol amendment that must be approved and signed by the sponsor, health authorities where required, and the IEC/REB.

# REFERENCES

1. Guan W, Ni Z, Hu Y, Liang W, Ou C, He J, et al. Clinical characteristics of coronavirus disease 2019 in China. *N Engl J Med* 2020; doi: 10.1056/ NEJMoa2002032
2. Huang C, Wang Y, Li X, Ren L, Zhao J, Hu Y, et al. [Clinical features of patients infected with 2019 novel coronavirus in Wuhan, China.](https://www.ncbi.nlm.nih.gov/pubmed/31986264) *Lancet* 2020; 395: 497-506
3. [Pixley RA](https://www.ncbi.nlm.nih.gov/pubmed/?term=Pixley%20RA%5BAuthor%5D&cauthor=true&cauthor_uid=21544310), [Espinola RG](https://www.ncbi.nlm.nih.gov/pubmed/?term=Espinola%20RG%5BAuthor%5D&cauthor=true&cauthor_uid=21544310), [Ghebrehiwet B](https://www.ncbi.nlm.nih.gov/pubmed/?term=Ghebrehiwet%20B%5BAuthor%5D&cauthor=true&cauthor_uid=21544310), [Joseph K](https://www.ncbi.nlm.nih.gov/pubmed/?term=Joseph%20K%5BAuthor%5D&cauthor=true&cauthor_uid=21544310), [Kao A](https://www.ncbi.nlm.nih.gov/pubmed/?term=Kao%20A%5BAuthor%5D&cauthor=true&cauthor_uid=21544310), [Bdeir K](https://www.ncbi.nlm.nih.gov/pubmed/?term=Bdeir%20K%5BAuthor%5D&cauthor=true&cauthor_uid=21544310), et al. Interaction of high-molecular-weight kininogen with endothelial cell binding proteins suPAR, gC1qR and cytokeratin 1 determined by surface plasmon resonance (BiaCore). [*Thromb Haemost*](https://www.ncbi.nlm.nih.gov/pubmed/21544310) 2011; 105: 1053-1059
4. [Schultz M](https://www.ncbi.nlm.nih.gov/pubmed/?term=Schultz%20M%5BAuthor%5D&cauthor=true&cauthor_uid=31236143), [Rasmussen LJH](https://www.ncbi.nlm.nih.gov/pubmed/?term=Rasmussen%20LJH%5BAuthor%5D&cauthor=true&cauthor_uid=31236143), [Høi-Hansen T](https://www.ncbi.nlm.nih.gov/pubmed/?term=H%C3%B8i-Hansen%20T%5BAuthor%5D&cauthor=true&cauthor_uid=31236143), [Kjøller E](https://www.ncbi.nlm.nih.gov/pubmed/?term=Kj%C3%B8ller%20E%5BAuthor%5D&cauthor=true&cauthor_uid=31236143), [Jensen BN](https://www.ncbi.nlm.nih.gov/pubmed/?term=Jensen%20BN%5BAuthor%5D&cauthor=true&cauthor_uid=31236143), [Lind MN](https://www.ncbi.nlm.nih.gov/pubmed/?term=Lind%20MN%5BAuthor%5D&cauthor=true&cauthor_uid=31236143), et al. Early discharge from the emergency department based on soluble urokinase plasminogen activator receptor (suPAR) levels: A TRIAGE III substudy. [*Dis Markers*](https://www.ncbi.nlm.nih.gov/pubmed/31236143) 2019; 2019: 3403549
5. Giamarellos-Bourboulis EJ, Norrby-Teglund A, Mylona V, Savva A, Tsangaris I, Dimopoulou I, et al. Risk assessment in sepsis: a new prognostication score by APACHE II score and serum soluble urokinase plasminogen activator receptor. *Crit Care* 2012; 16: R149
6. Conti P, Ronconi G, Caraffa A, Gallenga CE, Ross R, Frydas I, et al. [Induction of pro-inflammatory cytokines (IL-1 and IL-6) and lung inflammation by Coronavirus-19 (COVID-19 or SARS-CoV-2): anti-inflammatory strategies.](https://www.ncbi.nlm.nih.gov/pubmed/32171193) *J Biol Regul Homeost Agents* 2020; 34: doi: 10.23812/CONTI-E
7. Anakinra SmPC <https://www.ema.europa.eu/en/documents/product-information/kineret-epar-product-information_en.pdf>
8. Opal SM, Fisher CJ Jr, Dhainaut JF, Vincent JL, Brase R, Lowry SF, et al. [Confirmatory interleukin-1 receptor antagonist trial in severe sepsis: a phase III, randomized, double-blind, placebo-controlled, multicenter trial. The Interleukin-1 Receptor Antagonist Sepsis Investigator Group.](https://www.ncbi.nlm.nih.gov/pubmed/9233735) *Crit Care Med* 1997; 25: 1115-1122
9. Shakoory B, Carcillo JA, Chatham WW, Amdur RL, Zhao H, Dinarello CA, et al. [Interleukin-1 receptor blockade is associated with reduced mortality in sepsis patients with features of macrophage activation syndrome: reanalysis of a prior phase III trial.](https://www.ncbi.nlm.nih.gov/pubmed/26584195) *Crit Care Med* 2016; 44: 275-281
10. Stern A, Green H, Paul M, Vidal L, Leibovici L. Prophylaxis for Pneumocystispneumonia (PCP) in non-HIV immunocompromised patients. *Cochrane Database Syst Rev.* 2014 Oct; doi: 10.1002/14651858.CD005590.pub3.
11. Trimethoprim/sulfamethoxazole SmPC <https://www.medicines.org.uk/emc/product/5752/smpc>

# APPENDIX I List of study sites

- 1^st^ Department of Internal Medicine, AHEPA University General Hospital of Thessaloniki (PI: Simeon Metallidis, Associate Professor of Internal Medicine and Infectious Diseases)
- 2^nd^ University Department of Internal Medicine, IPPOKRATEION General Hospital of Athens (PI: Helen Sambatakou, Assistant Professor of Internal Medicine and Infectious Diseases
- 3^rd^ University Department of Internal Medicine, General Hospital of Chest Diseases of Athens I SOTIRIA, (PI: Garyfallia Poulakou, Assistant Professor of Internal Medicine)
- Department of Internal Medicine, General Hospital of Chest Diseases of Athens I SOTIRIA (PI: Aikaterini Argyraki, Senior Registar)
- COVID-19 Department, General Hospital of Attica SISMANOGLEIO-AMALIA FLEMING, (PI: Malvina Lada, Director of NHS)
- Department of Internal Medicine, I PAMMAKARISTOS Hospital, (PI: Ioannis Baraboutis, Director of NHS)
- Department of Internal Medicine, University General Hospital of Patras PANAGIA I VOITHIA, (PI: Charalambos Gogos, Professor of Internal Medicine)
- Department of Internal Medicine, University General Hospital of Larissa, (PI: George Dalekos, Professor of Internal Medicine)
- 2^nd^ Department of Internal Medicine, University General Hospital of Alexandroupolis, (PI: Periklis Panagopoulos, Assistant Professor of Internal Medicine)
- Department of Clinical Therapeutics, ALEXANDRA General Hospital of Athens, (PI: Evangelos Kostis, Director of NHS)
- Department of Infectious Diseases, General Hospital of Kerkira, (PI: Ilias Papanikolaou, Senior Registar)
- 1^st^ University Department of Internal Medicine, General Hospital of Athens LAIKO, (PI: Michael Samarkos, Associate Professor of Internal Medicine and Infectious Diseases)
- 1^st^ Department of Internal Medicine, General Hospital of Athens G. GENNIMATAS, (PI: Georgios Adamis, Director of NHS)
- 1^st^ Department of Internal Medicine, General University Hospital of Ioannina, (PI: Charalampos Milionis, Professor of Internal Medicine)

**Monitor** Dr. Antigoni Kotsaki, MD, PhD

e-mail: [scra@sepsis.gr](mailto:scra@sepsis.gr) tel: +30 694 66 37 164; +30 210 58 32 562

**Pharmacovigilance supervisor** Ms Areti Voulomenou, MEng, MSc

e-mail: [voulomenou@suschem.gr](mailto:voulomenou@suschem.gr) tel +30 210 82 52 510

# APPENDIX II Study visits

| **Visits days** | **Screening** | **1** | **2** | **3** | **4** | **5** | **6** | **7** | **8** | **9** | **10** | **14** |
| --- | --- | --- | --- | --- | --- | --- | --- | --- | --- | --- | --- | --- |
| Informed consent | x |  |  |  |  |  |  |  |  |  |  |  |
| Exclusion criteria | x |  |  |  |  |  |  |  |  |  |  |  |
| Inclusion criteria | x |  |  |  |  |  |  |  |  |  |  |  |
| suPAR measurement | x |  |  |  |  |  |  | x |  |  |  |  |
| Scoring of symptoms |  | x |  |  |  |  |  | x |  |  |  | x |
| SOFA score |  | x |  |  |  |  |  | x |  |  |  | x |
| Blood sampling |  | x |  |  |  |  |  | x |  |  |  |  |
| Anakinra administration |  | x | x | x | x | x | x | x | x | x | x |  |
| Trimethoprim/sulfamethoxazole  administration |  | x | x | x | x | x | x | x | x | x | x |  |
| Severe respiratory failure |  |  | x | x | x | x | x | x | x | x | x | x |
| AE/SAE |  | x | x | x | x | x | x | x | x | x | x | x |

# APPENDIX III The SOFA score

| **Variable** | **0 points** | **1 point** | **2 points** | **3 points** | **4 points** |
| --- | --- | --- | --- | --- | --- |
| PaO_2_/FiO_2_(mmHg) | ≥400 | <400 | <300 | <200 | <100 |
| Platelets (per mm^3^) | ≥150 | <150 | <100 | <50 | <20 |
| Hypotension | MAP≥ 70 mmHg | MAP<70 mmHg | Dobutamine whatever dose | Adrenaline≤0.1* or  Noradrenaline  ≤0.1* | Adrenaline>0.1* or  Noradrenaline >0.1* |
| Glasgow Coma Scale | 15 | 13-14 | 10-12 | 6-9 | <6 |
| Bilirubin (mg/dl) | <1.2 | 1.2-1.9 | 2.0-5.9 | 6.0-11.9 | ≥12 |
| Creatinine (mg/dl) or Urine output | <1.2 | 1.2-1.9 | 2.0-3.4 | 35-4.9 or <500ml/day | ≥5.0 or  <200ml/day |

*μg/kg/min

Each variable is scored between 0 and 4. The SOFA score is the sum of the score of each variable

# APPENDIX IV Scoring of respiratory symptoms

| **Symptom** | **Absent (score=0)** | **Mild (score=1)** | **Moderate (score=2)** | **Severe (score=3)** |
| --- | --- | --- | --- | --- |
| **Cough** | No cough or resolution (to pre-CAP levels) | Cough present but it does not interfere with subject’s usual daily activities | Cough present, frequent and it does interfere with some of the subject’s usual daily activities | Cough is present throughout the day and night; it limits most of the subject’s usual daily activities and sleep patterns |
| **Chest pain** | No chest pain or resolution of chest pain related to CAP | Chest pain present occasionally with deep breathing but it does not interfere with subject’s usual daily activities | Chest pain is present with normal breaths and it does interfere with the subject’s usual daily activities | Chest pain is present at rest and/or with shallow breathing; it limits most of the subject’s usual daily activities |
| **Shortness of breath (dyspnea)** | No shortness of breath or resolution (to pre-CAP Baseline) | Shortness of breath with strenuous activities only but it does not interfere with subject’s usual daily activities | Shortness of breath with usual activities and it does interfere with the subject’s usual daily activities | Shortness of breath with minimal exertion or at rest; it limits most of the subject’s usual daily activities |
| **Sputum** | No coughing up of phlegm/sputum or resolution (to pre-CAP Baseline) | Subject coughs up a small amount of phlegm/sputum | Subject coughs up a moderate amount of phlegm/sputum | Subject coughs up a large amount of phlegm/sputum |

# APPENDIX V Definition of severe respiratory failure

Presence of all of the following:

- pO_2_/FiO_2_ less than 150
- Need for mechanical or non-mechanical ventilation (CPAP)

**STATISTICAL ANALYSIS PLAN (5 October 2020)**

The study is powered for the primary endpoint making the hypothesis that 60% of patients with lower respiratory tract infection due to COVID-19 and suPAR≥6 ng/ml upon hospital admission will develop SRF. To decrease this to 45% with power of 90% at a 5% level of significance, two hundred sixty (260) patients need to be enrolled. Taking into consideration, the single-arm study design, 500 patients should be enrolled to make results more robust. Data will be analyzed for the intention-to-treat (ITT) population.

An interim analysis will be conducted after completion of the 30-day follow up of the first 130 patients, i.e. 50% of the number of patients needed to be enrolled as calculated by power analysis. Parallel comparators receiving standard-of-care treatment (SOC) will be used all patients hospitalized at the same time period in eight departments of Internal Medicine in tertiary hospitals of Athens who were participating in the registry of the Hellenic Sepsis Study Group without participating in the SAVE trial ([www.sepsis.gr](http://www.sepsis.gr)). Data and biosample collection is done in the SOC comparators after written informed consent. The same inclusion and exclusion criteria will apply in the parallel comparators under SOC as in patients participating in the SAVE trial. Data collection in these departments has been approved by the Ethics Committees of the participating hospitals. Propensity matching will be performed among comparators to the 130 patients enrolled in the SAVE trial, so as the 130 comparators will be fully comparable to all below criteria:

- Age
- Comorbidities, as expressed by the Charlson’s Comorbidity Index
- Admission severity scores namely pneumonia severity index (PSI), acute physiology and chronic health evaluation (APACHE) II score, SOFA score and WHO severity classification of COVID-19
- Co-administered treatment of azithromycin, hydroxychloroquine and dexamethasone

Primary outcome and binary secondary outcomes as well as serious adverse event and adverse event rates between the two groups will be expressed as percentages and will be compared by the Fisher exact test, calculating also the respective odds ratios. The time to an event will be compared between the two groups by survival analysis. Stepwise Cox regression analysis will be conducted for the primary outcome and 30-day mortality. Quantitative secondary outcomes will be expressed as median values with respective interquartile range and will be compared by the Mann-Whitney U test.

The cost of hospitalization will be measured by adding the itemized cost of stay, drugs, and interventions and will be expressed as median with respective interquartile range. Salaries of nurse and medical stuff will not be included. Cost comparison will be done by the Mann-Whitney U test.

Qualitative baseline characteristics will be expressed as frequencies and percentages and compared by the Fisher exact test. Quantitative baseline characteristics will be expressed as mean values with standard deviation if following a normal distribution or median values with quartiles if following linear distribution. Comparisons of quantitative baseline characteristics will be done by the Student’s t-test or by the Mann-Whitney U test respectively. Paired comparisons will be done by the Wilcoxon’s rank-signed test. Non-parametric correlations will be done according to Spearman.

Analysis will be conducted with SPSS Version 25.0. All P values will be two-sided. Any P value <0.05 will be considered as statistically significant.
